# Identification of Plant Phenolics from Paulownia tomentosa and Morus alba as Novel PPARγ Partial Agonists and Hypoglycemic Agents

**DOI:** 10.1021/acs.jafc.4c11398

**Published:** 2025-05-20

**Authors:** Jakub Treml, Jiří Václavík, Lenka Molčanová, Marie Čulenová, Scarlet Hummelbrunner, Cathrina Neuhauser, Verena M. Dirsch, Julian Weghuber, Karel Šmejkal

**Affiliations:** † Department of Molecular Pharmacy, 37748 Masaryk University, 612 00 Brno, Czech Republic; ‡ Department of Natural Drugs, Masaryk University, 612 00 Brno, Czech Republic; § Department of Pharmaceutical Sciences, University of Vienna, A-1090 Vienna, Austria; ∥ Center of Excellence Food Technology and Nutrition, 118508 University of Applied Sciences Upper Austria, 4600 Wels, Austria; ⊥ FFoQSI GmbH-Austrian Competence Centre for Feed and Food Quality, Safety and Innovation, 3430 Tulln, Austria

**Keywords:** diabetes mellitus, hypoglycemic, natural products, plant phenolics, PPARγ

## Abstract

The aim of our study was to determine the PPARγ agonism and hypoglycemic activity of natural phenolics isolated from Paulownia tomentosa and Morus alba. We started with a molecular docking preselection, followed by *in vitro* cell culture assays, such as PPARγ luciferase reporter gene assay and PPARγ protein expression by Western blot analysis. The ability of the selected compounds to induce GLUT4 translocation in cell culture and lower blood glucose levels in chicken embryos was also determined. Among the thirty-six plant phenolic compounds, moracin M showed the highest hypoglycemic effect in an *in ovo* experiment (7.33 ± 2.37%), followed by mulberrofuran Y (3.84 ± 1.34%) and diplacone (3.69 ± 1.37%). Neither moracin M nor mulberrofuran Y showed a clear effect on the enhancement of GLUT4 translocation or agonism on PPARγ, while diplacone succeeded in both (3.62 ± 0.16% and 2.4-fold ± 0.2, respectively). Thus, we believe that the compounds moracin M, mulberrofuran Y, and diplacone are suitable for further experiments to elucidate their mechanisms of action.

## Graphical abstract

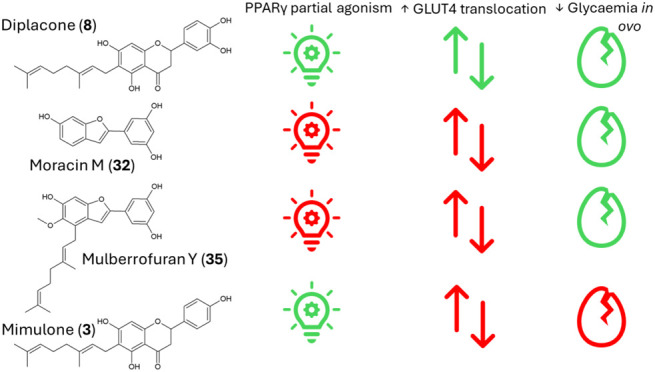

## Introduction

Diabetes mellitus (DM) is a group of endocrine disorders characterized by hyperglycemia. According to the International Diabetes Federation, 537 million adults had DM in 2021, and the number is expected to increase by 46% by 2045.^1^ There are two major types of DM – type 1, associated with insulin deficiency, and type 2 (T2DM), associated with insulin resistance manifesting as relative insulin insufficiency despite adequate insulin production. Uncontrolled hyperglycemia (usually in undiagnosed or uncooperative patients) leads to long-term complications such as diabetic nephropathy, retinopathy, and neuropathy.^2^ Treatment of T2DM, therefore, includes glucose-lowering medications along with general lifestyle changes to reduce body weight.^3^

Among oral antidiabetic agents, thiazolidinediones (TZDs) are highly effective and were first approved for treatment by the Food and Drug Administration (FDA) in the 1990s. A major boom in their prescription followed until 2005, but later their prescription gradually declined due to concerns about adverse effects, mainly the increased risk of heart attack and bladder cancer.^4,5^ The mechanism of action of TZDs is an agonistic effect on peroxisome proliferator-activated receptor γ (PPARγ), which leads to insulin sensitization.^4,5^

PPARγ is a member of the nuclear receptor family. Upon ligand binding, PPARγ transactivates specific target genes, thereby contributing to the regulation of glucose and lipid metabolism. The effects of PPARγ agonists are tissue-specific; in adipose tissue, they enhance adipogenesis, lipid metabolism, and the expression of the glucose transporter type 4 (GLUT4). The overall beneficial effect on T2DM is complemented by the production of adiponectin, which also increases glucose uptake in muscle cells.^5^ Today, partial PPARγ agonists that retain glucose-lowering benefits are considered as promising strategy to reduce the risk of adverse effects.^6^ Natural products from traditionally used medicinal plants have always been a promising pool of structures for drug discovery, and the field of PPARγ agonists and hypoglycemics is no exception.^4^

Paulownia tomentosa (Thunb.) Steud. (Paulowniaceae) is a deciduous tree and known in China as “Pao tong”.^7^ Its parts are widely used in Traditional Chinese Medicine (TCM) for the treatment of inflammation, which is a known condition associated with the development of T2DM.^8,9^ For example, tablets made from *Paulownia* leaves, fruit, and wood extracts are used to relieve cough and reduce mucus production in bronchitis.^10^ Morus alba L. (Moraceae) is also a deciduous tree known in TCM as “Sang”.^11^ Traditionally, an aqueous extract of M. alba leaves has been prescribed for the treatment of diabetes mellitus.^12^ In addition, mulberry fruit is commonly eaten fresh, dried, or processed as wine or juice. Its extracts have been associated with significant hypoglycemic activity.^13^ Both plants contain large amounts of plant phenolics, which are natural products containing aromatic rings substituted with one or more hydroxyl groups.^14,15^ Foods containing such compounds have health-protective functions that are relevant to the control of diet- and lifestyle-related chronic diseases, including T2DM. In addition to their beneficial antioxidant activity, many have been shown to have hypoglycemic effects.^16–18^

The aim of this study was to screen and determine the PPARγ agonism and hypoglycemic activity of thirty-six plant phenolic compounds. First, a preselection was performed by molecular docking to PPARγ, and second, the selected compounds were evaluated by *in vitro* cell culture-based PPARγ luciferase reporter gene assay and Western blot experiments to detect PPARγ protein expression. The ability of the selected compounds to induce GLUT4 translocation in cell culture and to lower blood glucose levels in chicken embryos was then determined.

## Materials and Methods

### Test Compounds

Eriodictyol (**1**) and naringenin (**2**) were used as nonprenylated flavanones for computational docking analysis together with compounds previously isolated from Paulownia tomentosa: 15 geranylated flavanones, mimulone (**3**),^19^ mimulone F (**4**),^20^ mimulone G (**5**),^20^ mimulone H (**6**),^20^ bonannione B (**7**),^20^ diplacone (**8**),^19^ 3′-*O*-methyl-5′-hydroxydiplacone (**9**),^21^ 3′-*O*-methyl-5′-*O*-methyldiplacone (**10**),^21^ tomentone II (**11**),^22^ tomentodiplacone G (**12**),^23^ tomentodiplacone L (**13**),^20^ tomentodiplacone M (**14**),^20^ tomentodiplacone N (**15**),^20^ tomentodiplacone O (**16**),^24^ and paulownione C (**17**);^24^ prenylated flavanonol 6-isopentenyl-3′-*O*-methyltaxifolin (**18**);^19^ caffeoyl phenylethanoid glycosides acteoside (**19**),^25^ isoacteoside (**20**);^25^ and the chromone 5,7-dihydroxy-6-geranylchromone (**21**)^26^ (Figure 1). Moreover, computational docking analysis was also performed with the geranylated flavanones kuwanon E (**22**),^27^ and kuwanon U (**23**);^27^ prenylated flavanone sanggenon H (**24**);^28^ three prenylated flavones, morusin (**25**),^29^ morusinol (**26**),^28^ and kuwanon C (**27**);^28^ four Diels–Alder adducts, sanggenon E (**28**),^28^ kuwanon K (**29**),^30^ kuwanon L (**30**),^30^ and kuwanon H (**31**);^30^ and five benzofurans, moracin M (**32**),^30^ moracin C (**33**),^30^ moracin O (**34**),^30^ mulberrofuran Y (**35**),^28^ and mulberrofuran H (**36**),^28^ all previously isolated from *Morus* sp. (Figure 1).

**Figure 1:**
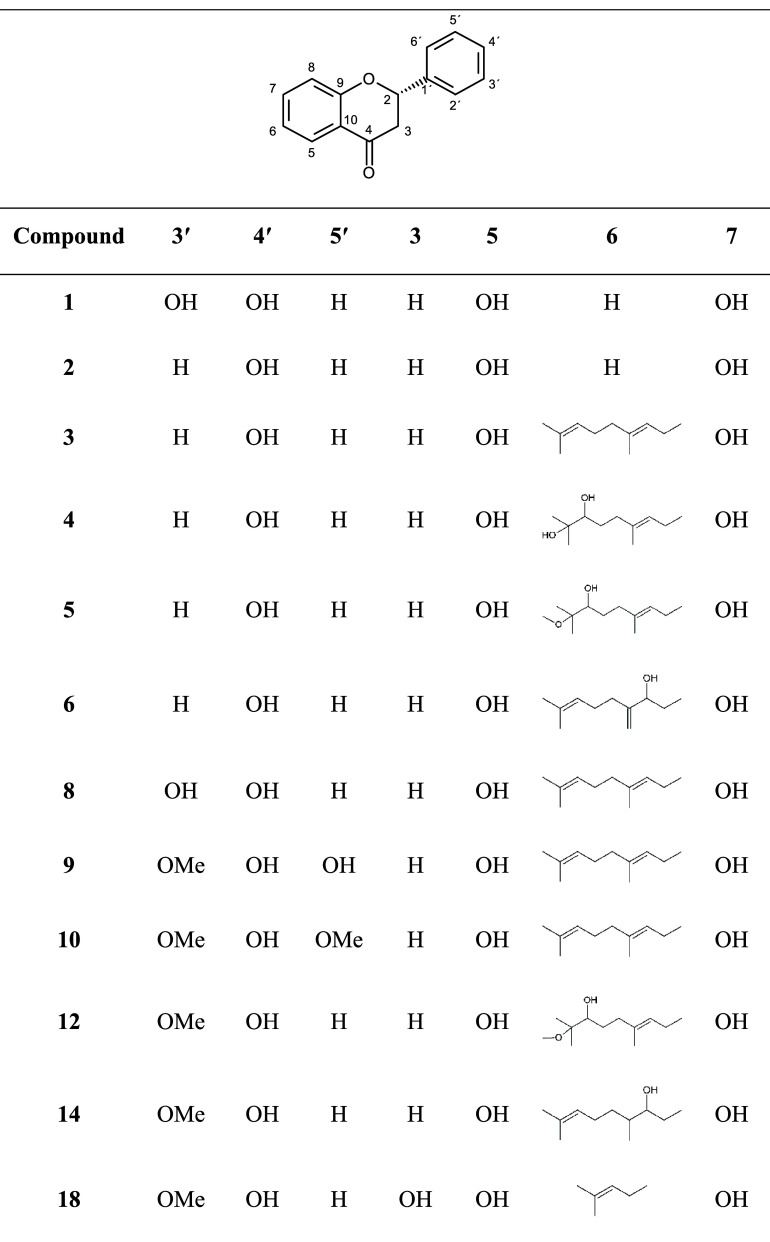

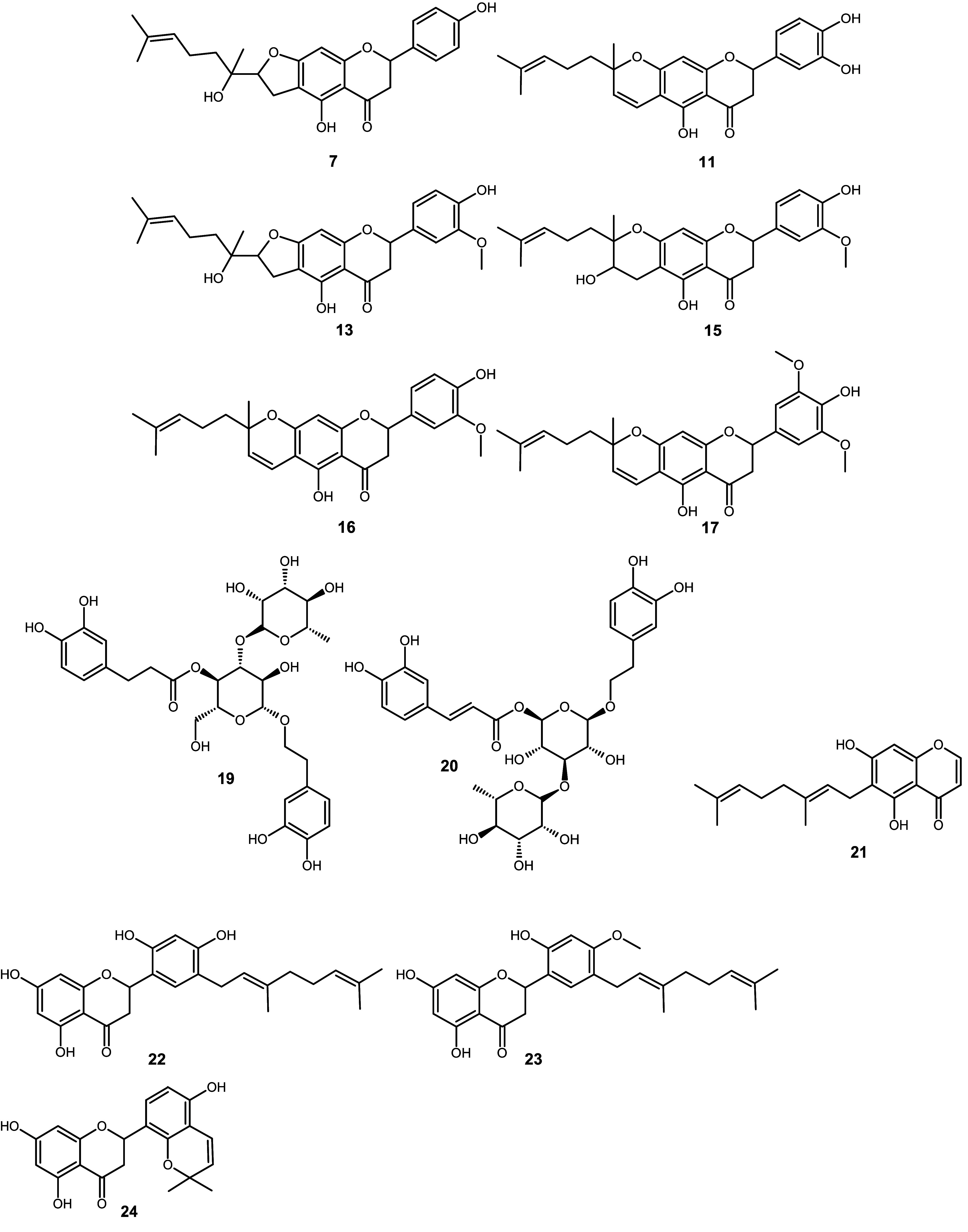

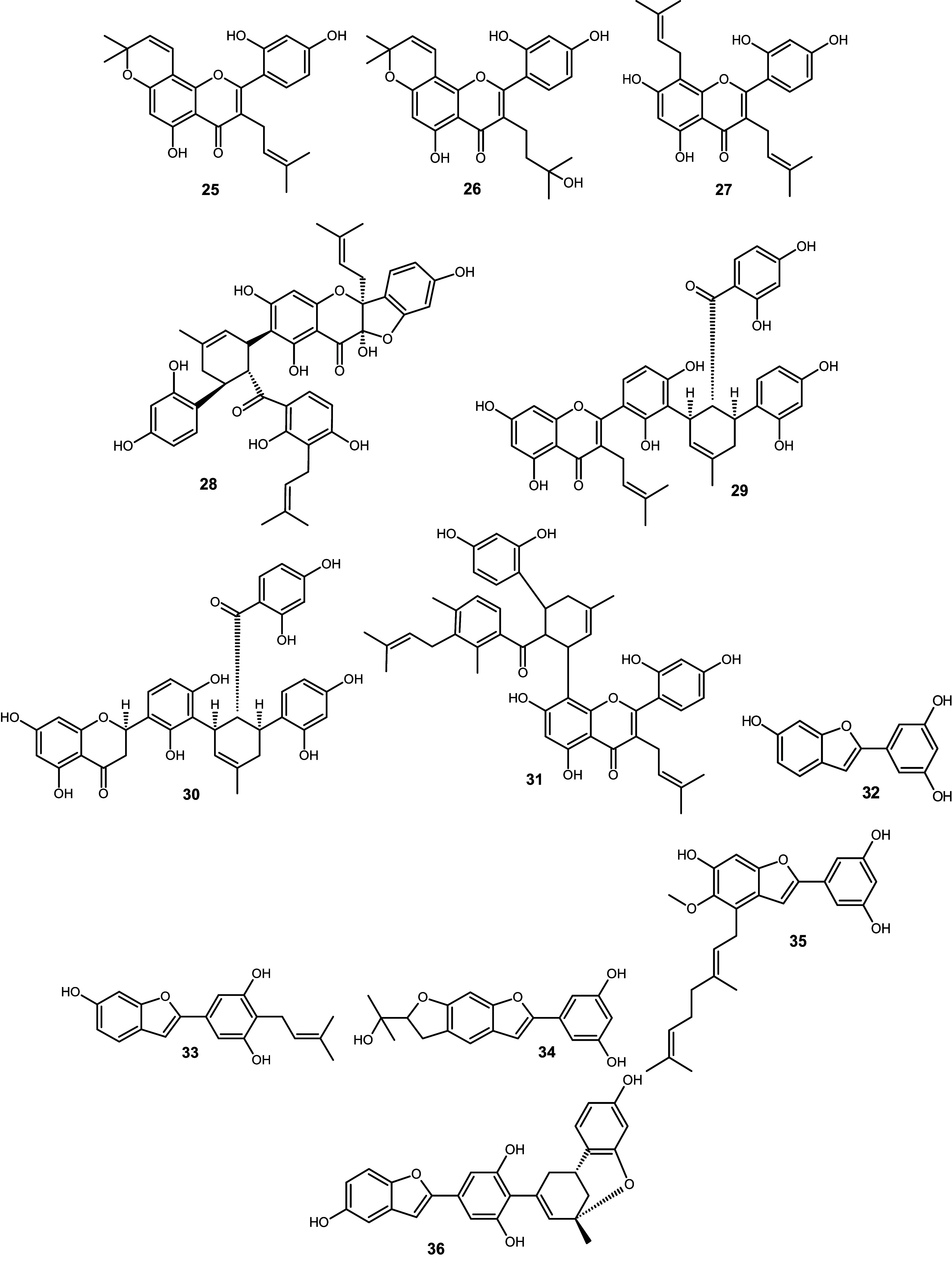
Compounds used for molecular docking: eriodictyol (**1**), naringenin (**2**), mimulone (**3**), mimulone F (**4**), mimulone G (**5**), mimulone H (**6**), bonannione B (**7**), diplacone (**8**), 3′-O-methyl-5′-hydroxydiplacone (**9**), 3′-O-methyl-5′-O-methyldiplacone (**10**), tomentone II (**11**), tomentodiplacone G (**12**), tomentodiplacone L (**13**), tomentodiplacone M (**14**), tomentodiplacone N (**15**), tomentodiplacone O (**16**), paulownione C (**17**), 6-isopentenyl-3′-O-methyltaxifolin (**18**), acteoside (**19**), isoacteoside (**20**), 5,7-dihydroxy-6-geranylchromone (**21**), kuwanon E (**22**), kuwanon U (**23**), sanggenon H (**24**), morusin (**25**), morusinol (**26**), kuwanon C (**27**), sanggenon E (**28**), kuwanon K (**29**), kuwanon L (**30**), kuwanon H (**31**), moracin M (**32**), moracin C (**33**), moracin O (**34**), mulberrofuran Y (**35**), mulberrofuran H (**36**).

### Isolation of Compounds for Bioactivity Assays

Compounds diplacone (**8**) and tomentone II (**11**) were obtained by the isolation procedures described in previous works. Briefly, the fruit of Paulownia tomentosa (Thunb.) Steud. (Paulowniaceae) was extracted with ethanol and further fractionated by liquid–liquid extraction into several portions. The methanol-soluble portion was subsequently separated by column chromatography using silica gel.^21^ Selected fraction *PT1Ia* was chosen for further separation using column chromatography and semipreparative RP-HPLC, leading to the isolation of compounds **8** and **11**.^22^

New plant material, 14.46 kg of the immature fruit of P. tomentosa, was collected in October 2021 and identified by Prof. PharmDr. Karel Šmejkal, Ph.D. The fruit was extracted with ethanol and the dried ethanolic extract (373.25 g) was further fractionated by liquid–liquid extraction into several portions. The chloroform portion (80.93 g) was subsequently separated by column chromatography using silica gel. Selected fraction *PT21/CH/A35–39+B31–39* was separated using semipreparative RP-HPLC, leading to the isolation of mimulone (**3**).

Kuwanon U (**23**) was obtained as described previously. Briefly, the dried root bark from Morus alba L. (Moraceae) was extracted with ethanol. Liquid–liquid extraction of the ethanol extract was carried out with chloroform and ethyl acetate. The chloroform soluble fraction was then separated using silica gel column chromatography and by semipreparative RP-HPLC, leading to the isolation of kuwanon U (**23**).^30^ Similarly, moracin C (**35**) and mulberrofuran Y (**33**) had been isolated previously from the chloroform-soluble fraction.^28^ The ethyl acetate-soluble material led to the isolation of moracin M (**32**) and moracin O (**34**).^30^

The test compounds used for the subsequent biological experiments were dissolved in dimethyl sulfoxide (DMSO); the final concentration of DMSO in the cellular assays was 0.1% (v/v). The purity of test compounds **3**, **8**, **11**, **23**, and **32** - **35** was confirmed by HPLC analysis to exceed 95% in all cases (for the methodology and results, see Supporting Information: Figures S1–S8).

### Molecular Docking

Molecular docking was done as reported previously.^31^ The crystal structure of the PPARγ receptor bound to rosiglitazone (Protein Data Bank (PDB) ID: 1FM6) was downloaded from the Collaboratory for Structural Bioinformatics PDB.^32^

PyRX was used in conjunction with AutoDock Vina. As a binding site, we used the position of rosiglitazone determined by crystallographic experiment. For the graphical evaluation of the results, PyMOL was used, and the best solution, ranked by binding affinity, was chosen.

### Chemicals, Cell Culture Reagents, and Plasmids

Fetal bovine serum (FBS), phosphate saline buffer (PBS), and Dulbecco’s modified Eagle’s medium (DMEM) were obtained from Lonza (Basel, Switzerland). All other chemicals were from Sigma-Aldrich (Vienna, Austria).

The PPAR luciferase reporter plasmid (tk-PPREx3-luc), pCMX-Gal4-hPPARγ, and tk­(MH1000)-4xLuc were a gift from Prof. Ronald M. Evans (Howard Hughes Medical Institute, La Jolla, CA),^33^ the plasmid encoding enhanced green fluorescent protein (pEGFP-N1) was from Clontech (Mountain View, CA), and the plasmid encoding human PPARγ (pSG5-PL-hPPAR-γ1) was a gift from Prof. Walter Wahli and Prof. Beatrice Desvergne (Center for Integrative Genomics, University of Lausanne, Switzerland).^34^

### PPARγ Luciferase Reporter Gene Transactivation

The PPARγ luciferase reporter gene transactivation experiments were done as reported previously.^35^ Briefly, human embryonic kidney HEK-293 cells (American Type Culture Collection (ATCC), Manassas, VA) were grown in DMEM supplemented with 2 mM l-glutamine, 100 U/mL benzylpenicillin, 100 μg/mL streptomycin, and 10% FBS. Cells were seeded in 10 cm dishes at a density of 6 × 10^6^ cells/dish for 18 h, and then transfected by the calcium phosphate precipitation method with 4 μg of PPARγ expression plasmid, 4 μg of reporter plasmid (tk-PPREx3-luc), and 2 μg of pEGFP-N1 used as internal control. Six hours later, cells were reseeded in 96-well plates (5 × 10^4^ cells/well) in DMEM without phenol red with 5% charcoal-stripped FBS, l-glutamine, and antibiotics. Cells were treated as indicated and incubated for 18 h. After cell lysis, the luminescence of the firefly luciferase and the fluorescence of EGFP were quantified on a GeniosPro plate reader (Tecan, Grödig, Austria). The luminescence signals were normalized to the EGFP-derived fluorescence to account for differences in cell number or transfection efficiency.

One-hybrid luciferase reporter system experiments on the PPARγ ligand binding domain (PPARγ-LBD) were done as reported previously.^36^ HEK-293 cells were transfected using the calcium phosphate method with the following plasmids: 6 μg of pCMX-Gal4-hPPARγ and 6 μg of tk­(MH1000)-4xLuc. Additionally, all cells were cotransfected with 3 μg of pEGFP-N1 to control transfection efficiency.

### Protein Expression

The effect of selected test compounds on PPARγ protein expression was measured on the HepG2 human hepatoma cell line, which was purchased from the European Collection of Cell Cultures (Salisbury, UK) and was cultured as reported previously.^37^ The HepG2 cells were incubated for 24 h with the selected test compounds.^38^ After the incubation, the cells were lysed and the lysates were processed using sodium dodecyl sulfate–polyacrylamide gel electrophoresis (SDS–PAGE) and Western blot as reported previously.^39,40^ Specific primary antibodies used for PPARγ: rabbit anti-PPARγ 1:1000 (Sigma-Aldrich; product No. SAB4502262).

### Total Internal Reflection Fluorescence (TIRF) Microscopy

HeLa cells stably expressing GLUT4-myc-GFP were maintained in RPMI 1640 cell culture medium supplemented with 100 μg/mL penicillin, 100 μg/mL streptomycin, 1% G418, and 10% fetal bovine serum (FBS) (M&B Stricker, Bernried, Germany) at 37 °C in a humidified atmosphere with 5% CO_2_. As previously reported,^41–43^ 40,000 HeLa GLUT4-myc-GFP cells/well were seeded into 96-well imaging plates (MoBiTec, Goettingen, Germany), grown overnight, washed twice with Hanks’ Balanced Salt solution (HBSS, Sigma-Aldrich, Schnelldorf, Germany), and starved for 3 h with HBSS. The cells were imaged using TIRF-Microscopy and stimulated with compounds **3**, **8**, **32**, and **35** (10 μM) and human insulin (100 nM) dissolved in KRPH buffer (pH = 7.4). Images were recorded at 10 min time intervals, before and after stimulation. TIRF-Microscopy was performed on an epi-fluorescence microscope (Nikon Eclipse Ti2, Tokyo, Japan) using a 60× CFI Plan-Apochromat objective. Scanning of multiple stage positions was supported by a motorized x-y stage (CMR-STG-MHIX2, Märzhäuser, Germany). Emission diode lasers (Toptica Photonics, Munich, Germany) were used for excitation of GFP at 488 nm and the fluorescence signal was recorded by an sCMOS camera (Zyla 4.2, Andor, Northern Ireland).

### Hen’s Egg Test-Chorioallantoic Membrane (HET-CAM)

The HET-CAM was used as reported previously.^44,45^ Briefly, eggs were incubated at 38 °C, 40–50% humidity for 11 days in an egg incubator (HEKA Brutgeräte, Rietberg, Germany). They were automatically and constantly turned. On the experimental day, the eggs were checked for fertilization via candling, and the air bladder area was marked. The eggshell was lightly pecked with a pointed pair of tweezers in this area and 300 μL of the Insulin Analog NovoRapid (3U/mL), which served as positive control and the compounds **3**, **8**, **32**, and **35** were applied with a syringe into the air compartment of the egg and incubated for an additional 1 to 2 h in the egg incubator. The compounds were dissolved in DMSO (Sigma-Aldrich) before being diluted in water and tested at a concentration of 40 μM. DMSO at 0.4% was applied to some eggs as an additional control besides water. After the incubation, the eggshell above the air bladder was carefully removed and the eggshell membrane was equilibrated with PBS (PAN-Biotech, Aidenbach, Germany). Following this, the eggshell membrane was removed and the chorioallantoic membrane was cut with a microscissor. A suitable blood vessel was placed on a plastic pH strip and patted dry using filter paper before being cut, and leaking blood was collected. The blood glucose levels were determined via a blood glucose meter (Accu-Check Performa, Roche Diabetes Care GmbH, Mannheim, Germany). For each time point, at least 10 fertilized eggs were used. The experiment was repeated three times.

### Statistical Evaluation

Statistical analyses were carried out using IBM SPSS Statistics for Windows software, version 26.0 (IBM, Armonk, NY). The data were graphed as the mean ± SEM. Comparisons between groups were made using a Kruskal–Wallis test, followed by a pairwise comparison with a Bonferroni correction or using a Mann–Whitney U test in the case of results of HET-CAM. Moreover, the data were compared using the calculation of Cohen’s *d* effect size.

## Results

### PPARγ Receptor Molecular Docking Study

The thirty-six test compounds were subjected to a PPARγ receptor molecular docking study. The known PPARγ agonist, rosiglitazone, was docked into the active site of the ligand binding domain (LBD) of the receptor to calculate the binding affinities of the test compounds in comparison to this positive control. The results are shown in Table 1. Rosiglitazone had a binding affinity of −8.8 kcal·mol^–1^. The test compounds had binding affinities ranging from −8.1 to −10.5 kcal·mol^–1^. The docking program identified three compounds from P. tomentosa (**3**, **8**, **11**) and five from M. alba (**23**, **32**-**35**) for further testing. The main criterion for the selection of the compounds from P. tomentosa was the binding energy (<−9.5 kcal·mol^–1^), since several other *Paulownia* compounds were able to fit into the active site of the ligand-binding domain (LBD). However, compounds **15** and **16** could not be isolated in sufficient quantities for *in vitro* biological assays. Only the five selected compounds from M. alba fit into the active site, and therefore, these were further analyzed regardless of the value of the binding energy.

**Table 1:** Binding Energy and Binding Location of the Test Compounds^a^

test compound	binding energy [kcal·mol^–1^]	ligand-binding domain
1	–8.3	yes
2	–8.3	yes
**3**	**–9.5**	**yes**
4	–9.2	yes
5	–8.5	yes
6	–8.4	yes
7	–9.3	yes
**8**	**–9.6**	**yes**
9	–9.3	yes
10	–9.3	yes
**11**	**–10.1**	**yes**
12	–8.7	yes
13	–9.8	no
14	–8.5	no
15	–9.9	yes
16	–9.7	yes
17	–9.3	no
18	–8.4	yes
19	–8.1	no
20	–8.9	no
21	–8.5	yes
22	–9.3	no
**23**	**–9.6**	**yes**
24	–9.1	no
25	–9.6	no
26	–9.2	no
27	–8.5	no
28	–9.1	no
29	–9.7	no
30	–9.5	no
31	–8.8	no
**32**	**–8.1**	**yes**
**33**	**–8.3**	**yes**
**34**	**–9.0**	**yes**
**35**	**–9.2**	**yes**
36	–10.5	no
**rosiglitazone**	**–8.8**	**yes**

a Compounds selected for subsequent in *vitro* experiments are shown in bold.

### PPARγ Luciferase Reporter Gene Transactivation

*In silico* hits were isolated from the respective natural sources and subjected to PPARγ-driven luciferase reporter gene assays. First, they were tested in HEK293 cells transiently transfected with the full-length receptor at a concentration of 1 μM. As shown in Figure 2, the most promising test compounds were compounds **3** and **8** isolated from P. tomentosa. In addition, test compounds **32** and **35** were selected for further experiments due to low (although not significant) activity among compounds isolated from M. alba and their different structural skeletons (i.e., benzofurans). All selected compounds were less active than rosiglitazone, which was chosen as a positive control.

**Figure 2:**
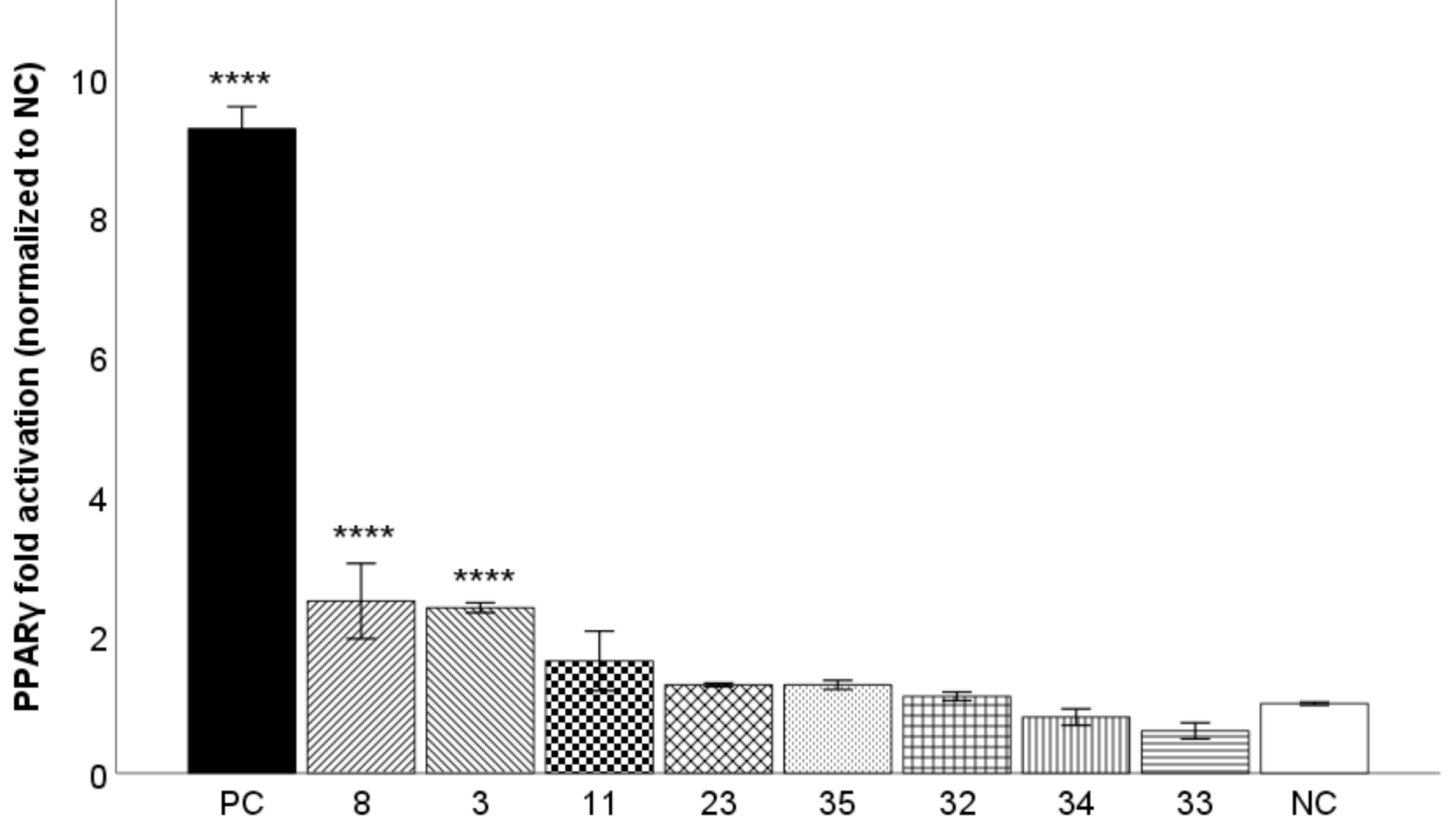
PPARγ agonistic activity of the test compounds from P. tomentosa (**3**, **8**, **11**) and M. alba (**23**, **32**-**35**) and rosiglitazone (PC) at a concentration of 1 μM after a 16 h incubation with the transiently transfected HEK293 cell line. The results are expressed as the mean ± SEM for three independent experiments measured in quadruplicate and are statistically compared to NC using a Kruskal–Wallis test followed by a pairwise comparison with a Bonferroni correction (**** *p* ≤ 0.0001).

Concentration–response curves were then performed with the selected compounds (**3**, **8**, **32**, and **35**). The test compounds were incubated with the same cell line at concentrations of 5, 3, 1, 0.3, and 0.1 μM. The most active compound was compound **3**, as shown in Figure 3. Compound **3** showed a statistically significant 3.4-fold activation of PPARγ at a concentration of 3 μM (*p* ≤ 0.0001; *d* = 7.431 (large)), whereas the positive control, rosiglitazone, activated PPARγ 7-fold (*d* = 6.612 (large)). Therefore, we hypothesized that compound **3** might be a partial agonist of PPARγ. Compound **8** showed a similar activity, although slightly lower, with 2.4-fold activation of the PPARγ receptor at a concentration of 3 μM (*p* ≤ 0.0001; *d* = 3.110 (large)). Compounds **32** and **35** activated PPARγ approximately 2-fold at concentrations of 3 and 5 μM.

**Figure 3:**
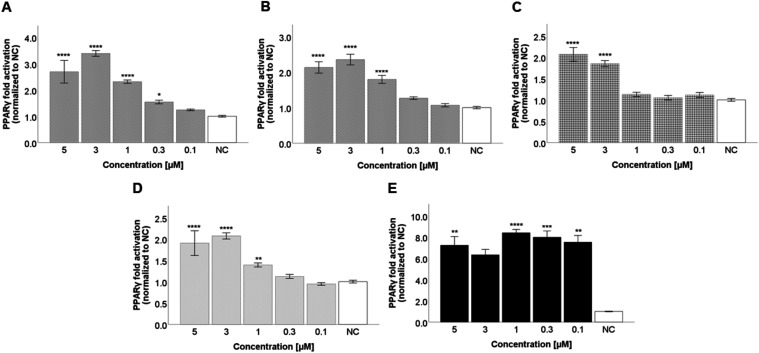
PPARγ agonistic activity of the test compounds **3** (A), **8** (B), **32** (C), **35** (D), and rosiglitazone (E). The test compounds were incubated with the transiently transfected HEK293 cell line at concentrations of 5, 3, 1, 0.3, and 0.1 μM. The results are expressed as the mean ± SEM for two independent experiments measured in quadruplicate and are statistically compared to NC using a Kruskal–Wallis test followed by a pairwise comparison with a Bonferroni correction (* *p* ≤ 0.05, ** *p* ≤ 0.01, *** *p* ≤ 0.001, and **** *p* ≤ 0.0001).

To further confirm PPARγ agonism, we employed a one-hybrid luciferase reporter system, in which the PPARγ ligand binding domain (PPARγ-LBD) is coupled to the DNA-binding domain of the yeast transcription factor Gal4, which then binds to its response element in the promoter of a luciferase reporter gene. The results for the same test compounds and concentrations are shown in Figure 4. Again, the highest activity was shown by compound **3** at a concentration of 5 μM (3.2-fold; *p* ≤ 0.0001; *d* = 8.345 (large)), followed by compound **8** (2.9-fold; *p* ≤ 0.0001; *d* = 2.820 (large)). Compound **35** also showed a small but significant activation of PPARγ at concentrations of 3 and 5 μM.

**Figure 4:**
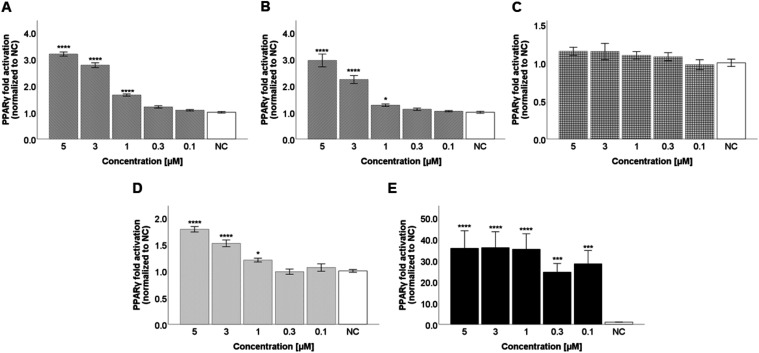
PPARγ-LBD agonistic activity (one-hybrid reporter assay) of the test compounds **3** (A), **8** (B), **32** (C), **35** (D), and rosiglitazone (E). The test compounds were incubated with the same cell line at concentrations of 5, 3, 1, 0.3, and 0.1 μM. The results are expressed as the mean ± SEM for three independent experiments measured in quadruplicate and are statistically compared to NC using a Kruskal–Wallis test followed by a pairwise comparison with a Bonferroni correction (** *p* ≤ 0.01, *** *p* ≤ 0.001, and **** *p* ≤ 0.0001).

Figures 5 and 6 visualize the docking of the two most promising compounds (**3** and **8**, respectively) into the active site of the LBD. Both compounds are shown in red and compared to rosiglitazone (blue). The results show that compound **3** fits into the pocket of the active site slightly better than compound **8**, which is consistent with the results shown in Figures 3 and 4.

**Figure 5:**
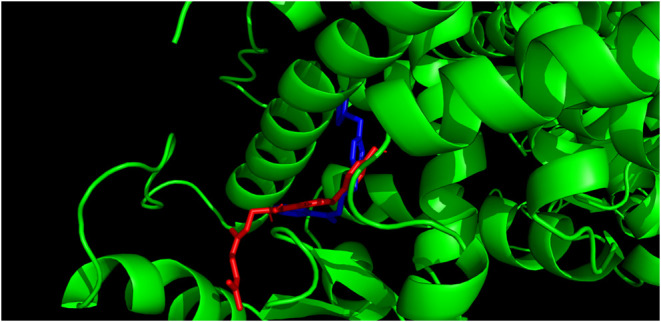
Visualization of molecular docking of compound **3** (red) into the active site of LBD compared to rosiglitazone (blue).

**Figure 6:**
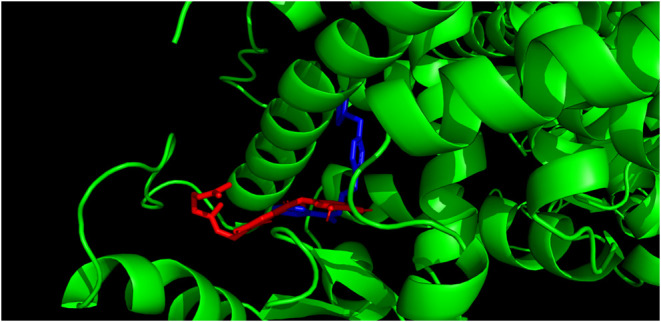
Visualization of molecular docking of compound **8** (red) into the active site of LBD compared to rosiglitazone (blue).

### PPARγ Protein Expression

The next step in our experiments was to determine whether the selected compounds (**3**, **8**, **32**, and **35**) were able to increase the level of PPARγ protein expression. The selected compounds were incubated with HepG2 cells at a concentration of 1 μM for 24 h and Western blot analyses were performed. None of the compounds showed a positive effect on PPARγ expression (data not shown) except compound **32**. As shown in Figure 7, compound **32** showed a tendency to increase the expression of PPARγ protein level, but without statistical significance.

**Figure 7:**
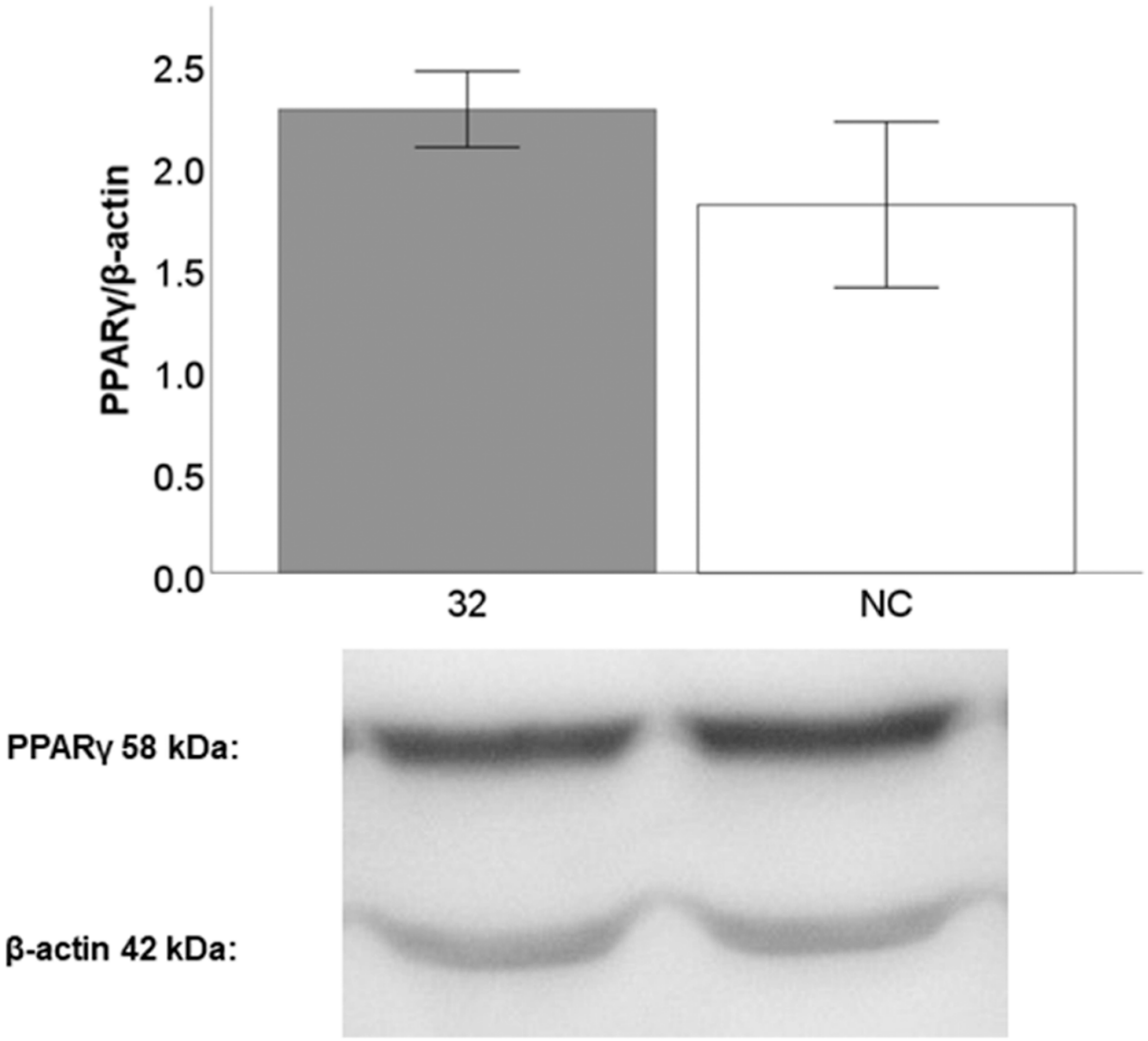
PPARγ protein expression after a 24-h incubation with compound **32** at a concentration of 1 μM, compared to DMSO used as solvent (NC). The results are expressed as the mean ± SEM for one experiment measured in triplicate.

### Quantification of GLUT4 Translocation

The selected compounds **3**, **8**, **32**, and **35** at a concentration of 10 μM were then tested for their ability to enhance GLUT4 translocation in HeLa GLUT4-myc-GFP cells using TIRF microscopy (Figure 8).^46^ The most active compound was compound **8**. After 10 min of incubation, the GFP signal in the cells increased by 3.62% in a statistically significant manner (*p* ≤ 0.0001; *d* = 4.545 (large)), which is approximately one-third of the activation of the positive control insulin at a concentration of 100 nM. Compound **3** was less active; in this case, the signal increased by 3.00% (*p* ≤ 0.0001). On the other hand, the remaining compounds **32** and **35** slightly increased the signal after 10 min (*p* ≤ 0.0001), but their GLUT4-GFP signal change values were below the 3% threshold for positive results.^41,42,47^

**Figure 8:**
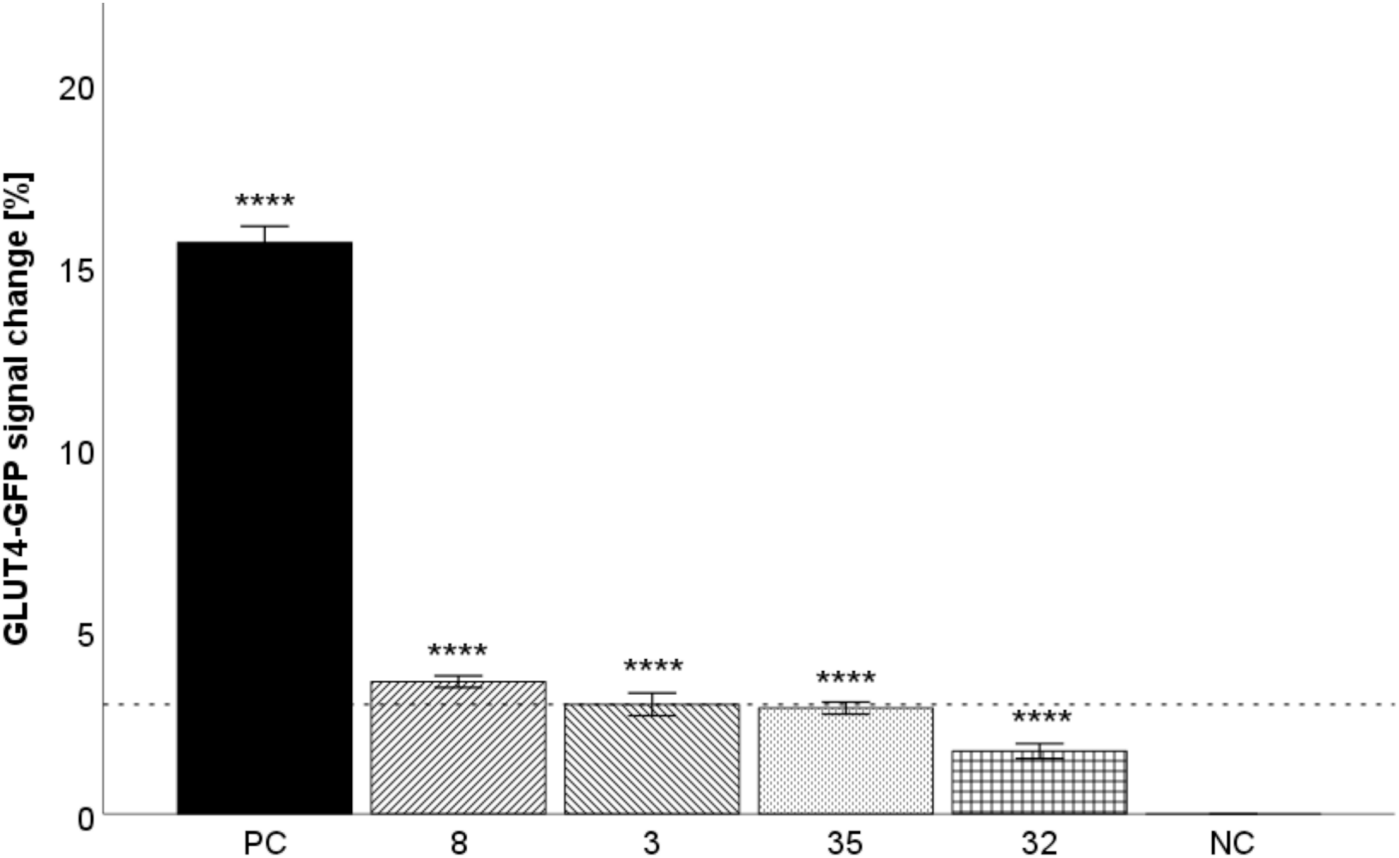
Quantification of GLUT4 translocation in HeLa GLUT4-myc-GFP cells after incubation with the test compounds **3**, **8**, **32**, and **35** at a concentration of 10 μM. Insulin (100 nM) was used as a positive control (PC). KRPH was used as a negative control (NC). The GLUT4-myc-GFP signal change was analyzed, and a threshold of 3% was defined for positive hits (dashed line).^41,42,47^ Data are shown as the mean ± SEM (*n* > 42) and are statistically compared to NC using a Kruskal–Wallis test followed by a pairwise comparison with a Bonferroni correction (** *p* ≤ 0.01, and **** *p* ≤ 0.0001).

### Hen’s Egg Test-Chorioallantoic Membrane (HET-CAM)

Finally, the selected compounds (**3**, **8**, **32**, and **35**) were evaluated for their efficacy in lowering blood glucose levels in chicken embryos using the HET-CAM assay. The selected compounds (at a concentration of 40 μM) were incubated for 60 and 120 min to observe possible glucose-lowering effects. An insulin analog (3 U/mL) was used as a positive control. The data for the change in blood glucose levels normalized to the solvent (0.4% DMSO) are shown in Figure 9. Compound **32** proved to be the most potent with a statistically significant 7.33% reduction in blood glucose after 120 min (*p* ≤ 0.01; *d* = 0.931 (large)). Two other compounds, **8** and **35**, were able to statistically significantly reduce blood glucose levels by 3.69 and 3.84% after 120 min (*p* ≤ 0.01; *d* = 0.741 (medium/large) and *p* ≤ 0.001; *d* = 0.836 (large)), respectively, while compound **3** showed a negligible effect.

**Figure 9:**
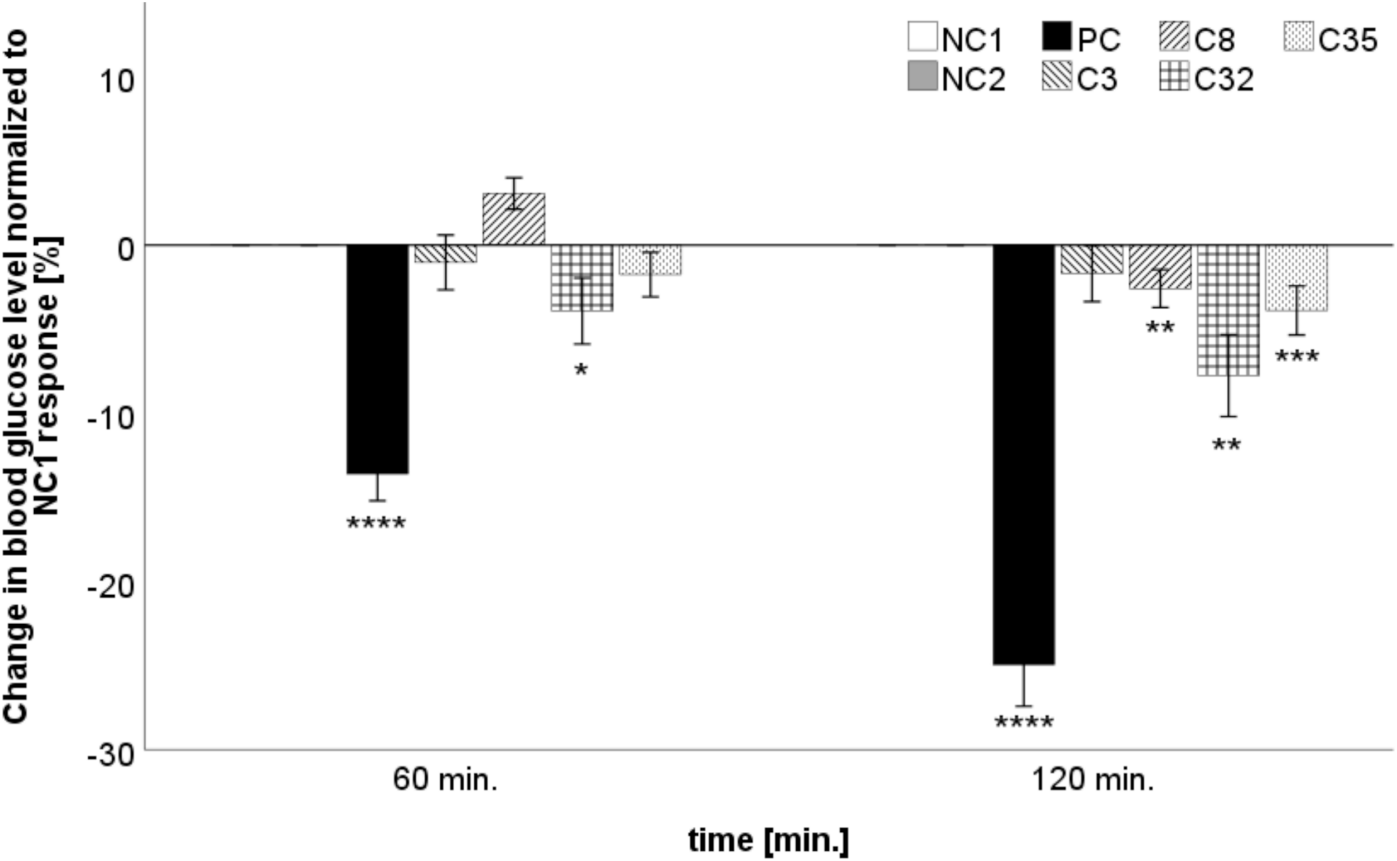
Quantification of lowering blood glucose efficacy in chicken embryos using HET-CAM assay. The test compounds (**3**, **8**, **32**, and **35**) at a concentration of 40 μM were incubated for 60 and 120 min to observe possible glucose-lowering effects. The insulin analog NovoRapid (3U/ml) was used as a positive control (PC). The data for the change in blood glucose levels were normalized to the solvent (0.4% DMSO; NC2), are shown as the mean ± SEM (*n* = 23–28), and are statistically compared to ultrapure water using a Mann–Whitney U test (NC1; * *p* ≤ 0.05, ** *p* ≤ 0.01, *** *p* ≤ 0.001, and **** *p* ≤ 0.0001).

## Discussion

Based on the data obtained from our experiments, mimulone (**3**) can be described as a partial PPARγ agonist. It did not influence PPARγ expression, slightly affected the translocation of GLUT4 transporters and insignificantly decreased glucose levels during *in ovo* experiments. Diplacone (**8**) showed a similar profile of activity, with the exception of a greater effect on GLUT4 translocation and a significant decrease in glycemia *in ovo*. Both compounds are geranylated flavanones with proven antioxidant and anti-inflammatory activity.^48,49^

Consistent with our observations, Zhang et al. described how compound **1** (structurally similar to diplacone (**8**), but without the geranyl moiety) increased insulin-stimulated glucose uptake in both HepG2 and differentiated 3T3-L1 adipocytes under high-glucose conditions. This compound was also able to upregulate PPARγ2 expression at both the mRNA and protein levels. Furthermore, compound **1** was able to reactivate an important kinase, protein kinase B (Akt), in the insulin signaling pathway in HepG2 cells with high-glucose-induced insulin resistance.^50^

Another step in reinforcing the insulin pathway may be the inhibition of protein tyrosine phosphatase 1B (PTP1P). Song et al. demonstrated on isolated enzyme that mimulone (**3**) was the most active with IC_50_ 1.9 μM.^51^ Diplacone (**8**) was not among the compounds tested in their experiment, but from the results, we can deduce that the most preferred arrangement of ring B of the flavonoid is with the hydroxyl at C-4′. Other hydroxy or methoxy groups at C-3′ or C-5′ decreased the activity.

Zima et al. performed *in vivo* experiments on alloxan-induced diabetes in mice to demonstrate the antidiabetic activity of compounds **3** and **8**.^52^ Their results do not fully agree with the results of our experiments. Compounds **3** and **8** were not able to reduce blood glucose levels in their model, with the only exception that compound **8** showed a significant reduction on day 1 (*p* ≤ 0.05). On the other hand, Zima et al. found a cytoprotective effect of compound **8** on pancreatic cells, which may be explained by its antioxidant activity, since alloxan induces diabetes via oxidative damage to the pancreas.^52^

Moracin M (**32**) and mulberrofuran Y (**35**) showed very low agonism to PPARγ compared to the two previous compounds. In GLUT4 translocation experiments, both compounds significantly induced translocation, but their GLUT4-GFP signal change values are below the 3% threshold for positive results. Both **32** and **35** were active in the *in ovo* blood glucose assays, with **32** being the most active among the compounds tested, significantly lowering blood glucose levels, especially after 60 min of incubation. The efficacy obtained was comparable to that of various plant extracts tested in several previous studies.^47,53,54^

Moracin M (**32**) is not described as a PPARγ agonist in the scientific literature, but a similar compound, moracin D, is. Compared to compound **32**, the structure of moracin D contains an additional prenyl moiety that is condensed with the hydroxyl group on the aryl segment of the structure. Moracin D was able to activate PPARγ/PKC-δ and inhibit PKC-α, thereby inducing apoptosis in prostate cancer DU145 cells.^55^

Kwon et al. reported PTP1B inhibitory activity for compound **32**, but this activity was very low, with an IC_50_ of 333.1 ± 20.53 μM.^56^ Consistent with our results, Zhang et al. reported a decreasing trend in fasting blood glucose levels in alloxan-diabetic mice after administration of compound **32**. The effect was dose-dependent, and the fasting blood glucose level was 18.52 ± 6.61 mmol/L at the dose of 100 mg/kg, which was not significantly different from that of the model group (23.58 ± 5.61 mmol/L).^57^ The possible mechanism of the hypoglycemic effect of moracin M (**32**) was described by Kwak et al.^58^ Moracin M (**32**) incubated with C2C12 cells (at concentrations of 5 and 25 μM) induced phosphorylation of phosphatidylinositol 3-kinase (PI3K) and Akt, two key regulators in the insulin transduction pathway.^58^

Also, mulberrofuran Y (**35**) has not been previously reported as a PPARγ agonist or as a hypoglycemic agent. However, another compound from the group of 2-arylbenzofurans, mulberrofuran G, has been reported as an inhibitor of PTP1B, with an IC_50_ of 0.57 ± 0.04 μM.^59^ Ha et al. also reported the same compound as an inhibitor of PTP1B, but with a different IC_50_ value of 4.56 ± 0.88 μM. In the same work, other compounds of our study (**27**, **32**, **34**, and **36**) were screened for PTP1B inhibition, but their effect was negligible.^60^

The bioavailability and intestinal absorption of the tested compounds are not known. However, Erlund et al. studied the plasma kinetics of the flavanones naringenin (**2**) and hesperetin (both similar to compound **8**) in humans. After ingestion of orange or grapefruit juice (8 mL/kg), the peak plasma concentration reached 6.0 ± 5.4 μM for naringenin (**2**) and 2.2 ± 1.6 μM for hesperetin.^61^ In addition, You et al. administered moracin C (similar to compounds **32** and **35**) orally to mice (100 mg/kg) and the peak plasma concentration was 5.8 ± 4.0 μM.^62^ These results are quite variable, but the concentrations we used in our experiments are achievable.

The three main tissues in the human body involved in the regulation of glycemia are adipose tissue, liver, and skeletal muscle. The HeLa cell line used in our GLUT4 translocation experiment is derived from human cervical carcinoma cells but is also insulin responsive (Figure 8). The HET-CAM experiments take advantage of the use of chicken embryos at day 11 of development. This avoids the need for animal experiments, which require approval, and the model has the advantage of mimicking *in vivo* conditions.^45^

In summary, the selected compounds showed a diverse profile of action as hypoglycemic agents. Our hypothesis of selecting compounds based on PPARγ agonism and subsequent investigation of their effect on GLUT4 translocation and glycemia *in ovo* appeared not to be generally useful. Moracin M (**32**) and mulberrofuran Y (**35**) reduced glycemia *in ovo* without convincing agonism on PPARγ, whereas mimulone (**3**) was shown to be a partial PPARγ agonist without affecting glycemia *in ovo*. Only diplacone (**8**) showed activity in all these aspects.

## Supplementary Material



## Notes

HPLC analysis of test compounds; HPLC chromatogram of mimulone (**3**) (Figure S1); HPLC chromatogram of diplacone (**8**) (Figure S2); HPLC chromatogram of tomentone II (**11**) (Figure S3); HPLC chromatogram of kuwanon U (**23**) (Figure S4); HPLC chromatogram of moracin M (**32**) (Figure S5); HPLC chromatogram of mulberrofuran Y (**33**) (Figure S6); HPLC chromatogram of moracin O (**34**) (Figure S7); HPLC chromatogram of moracin C (**35**) (Figure S8) (PDF)

## Abbreviations

ATCC: American Type Culture Collection
DM: diabetes mellitus
DMEM: Dulbecco’s modified Eagle’s medium
DMSO: dimethyl sulfoxide
DNA: deoxyribonucleic acid
EGFP: enhanced green fluorescent protein
FBS: fetal bovine serum
FDA: Food and Drug Administration
GLUT4: glucose transporter type 4
HBSS: Hanks’ Balanced Salt solution
HEK: human embryonic kidney
HET-CAM: hens egg test-chorioallantoic membrane
LBD: ligand-binding domain
PBS: phosphate saline buffer
PDB: Protein Data Bank
PKC: protein kinase C
PPARγ: peroxisome proliferator-activated receptor γ
PTP1B: protein tyrosine phosphatase 1B
SDS-PAGE: sodium dodecyl sulfate–polyacrylamide gel electrophoresis
T2DM: type 2 diabetes mellitus
TIRF: total internal reflection fluorescence
TZD: thiazolidinediones
