# Supplementary material for: Identification of Plant Phenolics from Paulownia tomentosa and Morus alba as Novel PPARγ Partial Agonists and Hypoglycemic Agents
Source: J Agric Food Chem. 2025 May 20;73(22):13960–72. doi: 10.1021/acs.jafc.4c11398 (PMC12147210; doi:10.1021/acs.jafc.4c11398)
Supplement: Supplementary file 1 [file jf4c11398_si_001.pdf]

Supporting Information

Identification of Plant Phenolics from *Paulownia tomentosa* and *Morus alba* as Novel PPARγ Partial Agonists and Hypoglycemic Agents

Jakub Treml[a*], Jiří Václavík[b], Lenka Molčanová[b], Marie Čulenová[b], Scarlet Hummelbrunner[c], Cathrina Neuhauser[d], Verena M. Dirsch[c], Julian Weghuber[d,e], Karel Šmejkal[b]

[a]Department of Molecular Pharmacy, Masaryk University, 612 00 Brno, Czech Republic

[b]Department of Natural Drugs, Masaryk University, 612 00 Brno, Czech Republic

[c]Department of Pharmaceutical Sciences, University of Vienna, A-1090 Vienna, Austria

[d]Center of Excellence Food Technology and Nutrition, University of Applied Sciences Upper Austria, 4600 Wels, Austria

[e]FFoQSI GmbH-Austrian Competence Centre for Feed and Food Quality, Safety and Innovation, 3430 Tulln, Austria

*** Correspondence:**

Jakub Treml

E-mail address: tremlj@pharm.muni.cz

HPLC analysis of test compounds. Analytical HPLC measurements were carried out with an Agilent 1100 chromatographic system (Agilent Technologies). The purity of the isolated compounds was evaluated using HPLC-DAD analysis (10–100% MeCN, 90-0% 0.2 HCOOH; 36 min; 0.3 mL/min; Ascentis Express RP-amide 100×2.1 mm, 2.7 μm (Supelco); 40°C), with monitoring of 230, 254, 280, and 350 nm, respectively, and exceeded 95% in all cases.

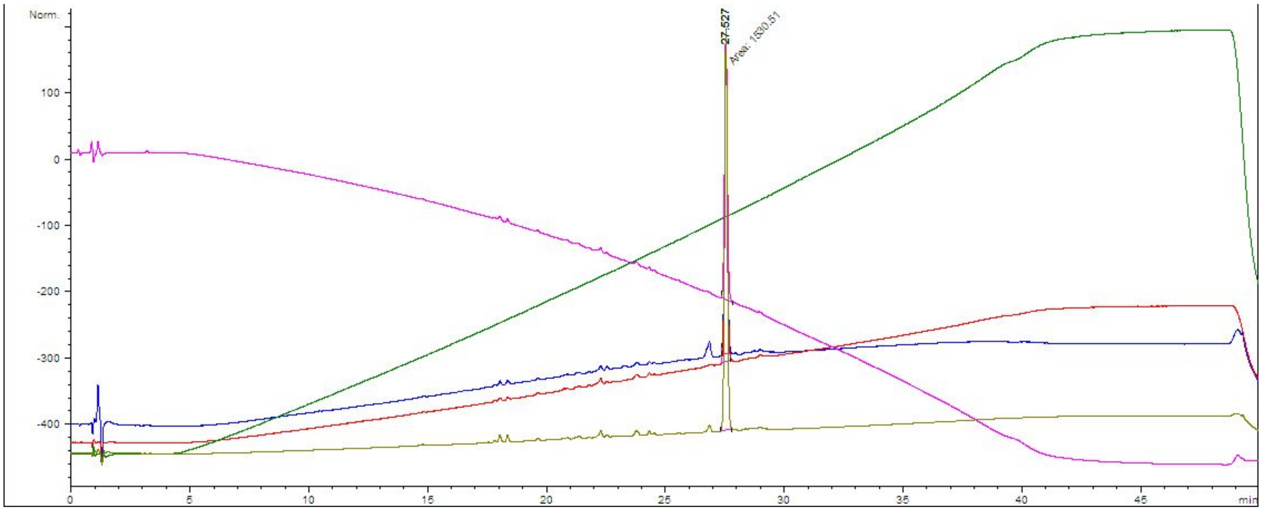

Figure S1. HPLC chromatogram of mimulone (**3**) at λ 254, 280, 350, and 230 nm.

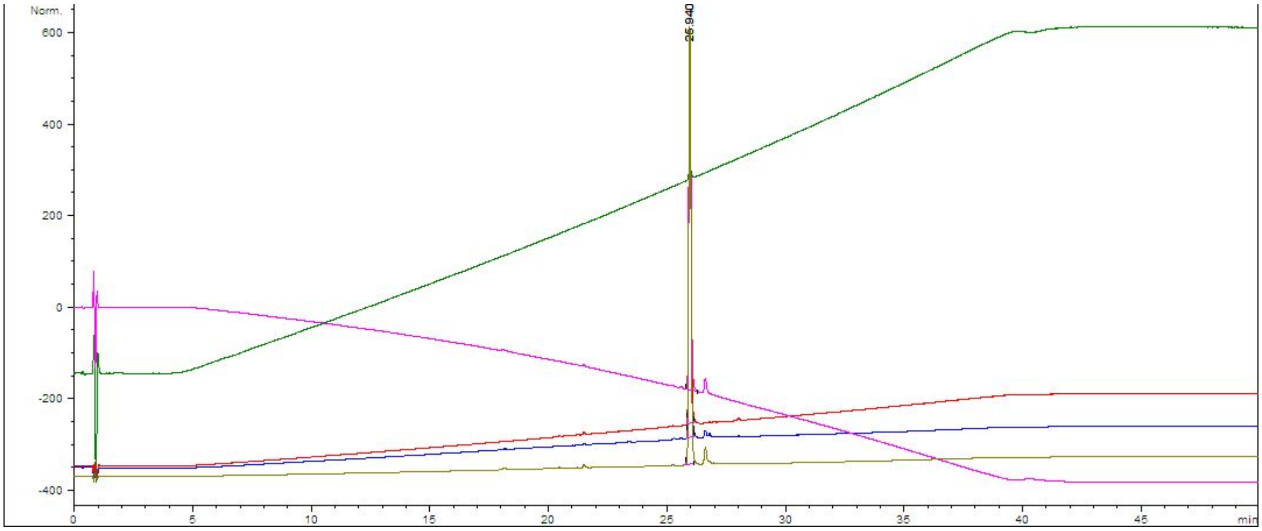

Figure S2. HPLC chromatogram of diplacone (**8**) at λ 254, 280, 350, and 230 nm.

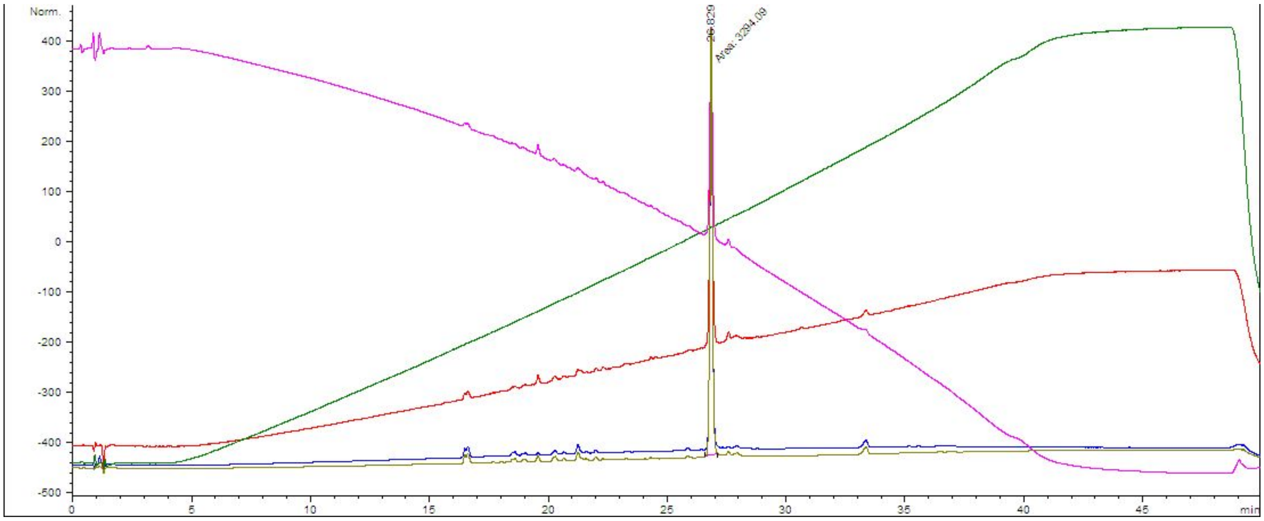

Figure S3. HPLC chromatogram of tomentone II (**11**) at λ 254, 280, 350, and 230 nm.

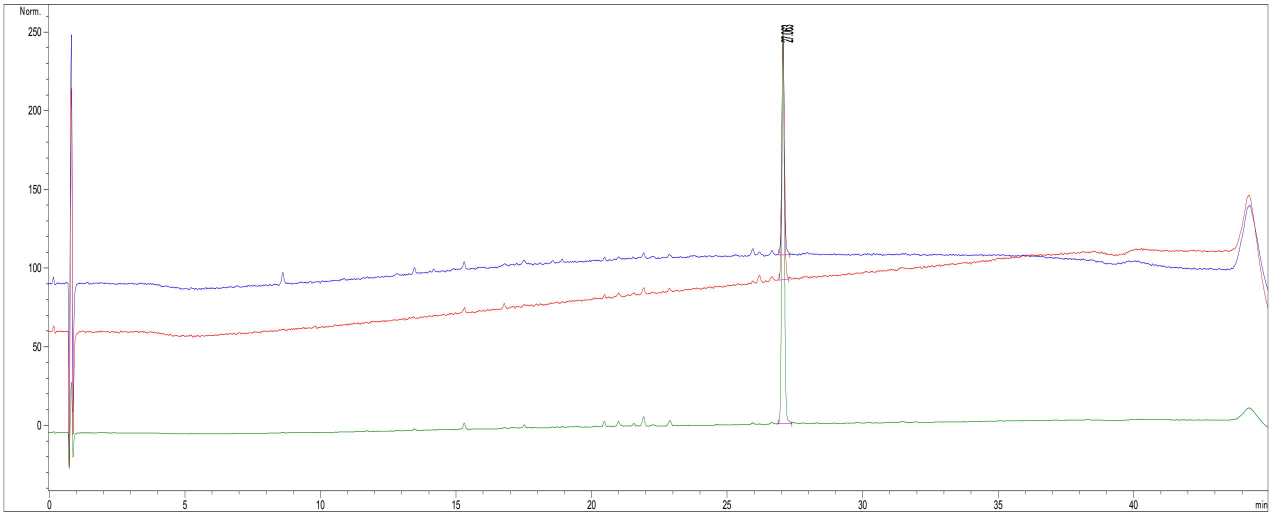

Figure S4. HPLC chromatogram of kuwanon U (**23**) at λ 254, 280, 350, and 230 nm.

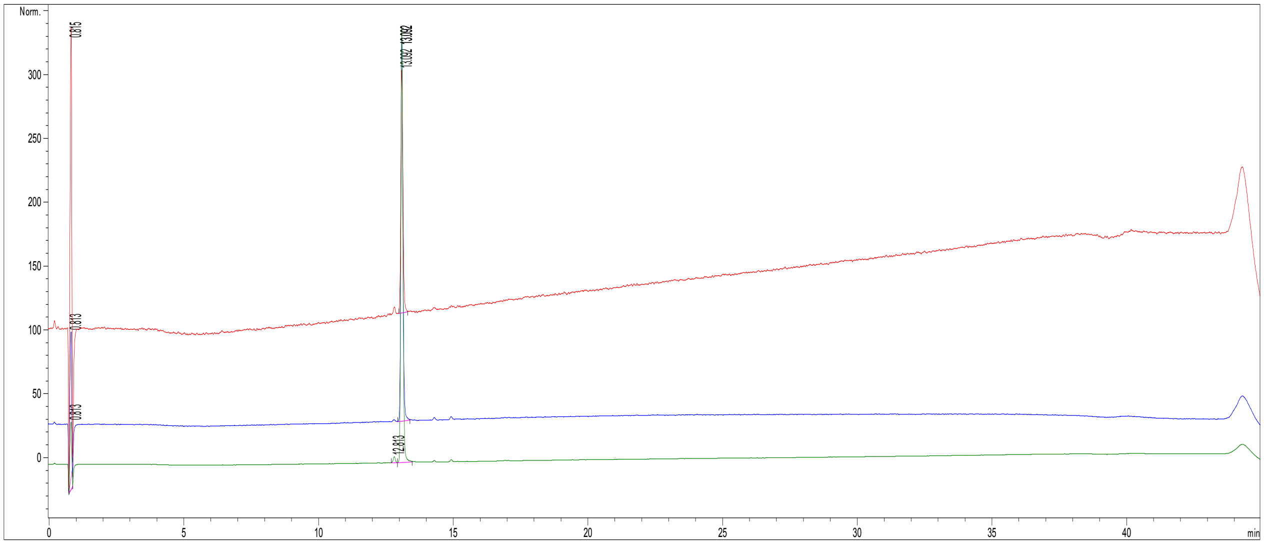

Figure S5. HPLC chromatogram of moracin M (**32**) at λ 254, 280, 350 nm.

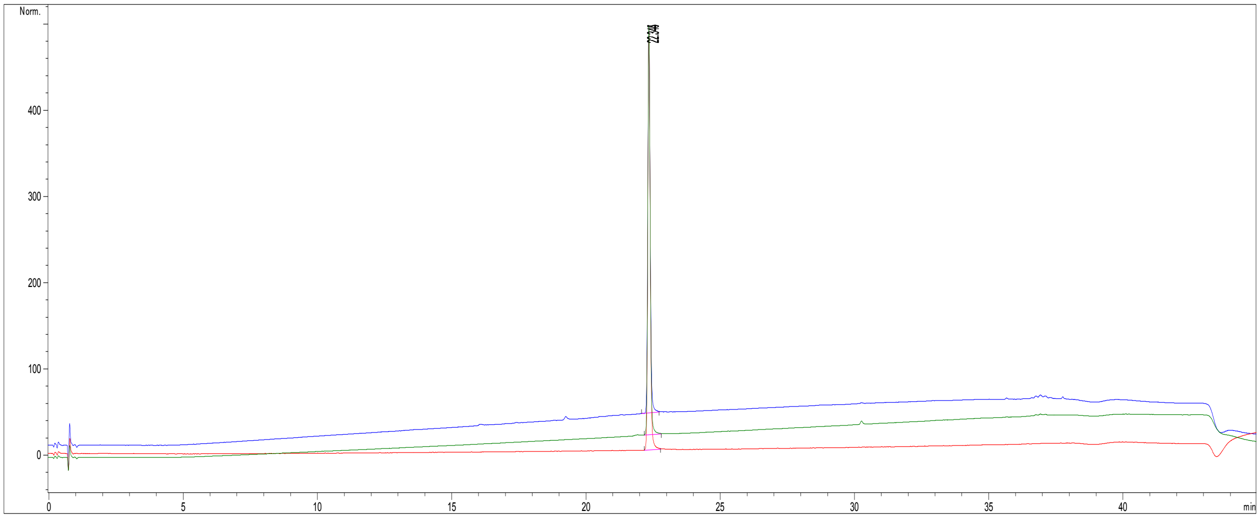

Figure S6. HPLC chromatogram of mulberrofuran Y (**33**) at λ 254, 280, 350 nm.

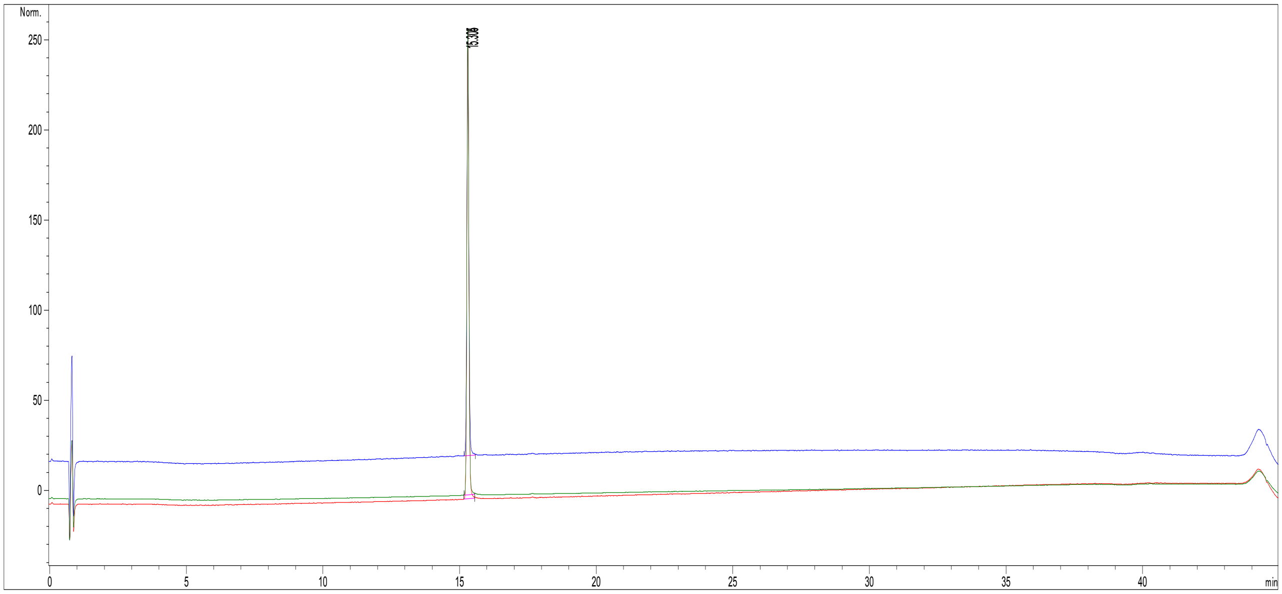

Figure S7. HPLC chromatogram of moracin O (**34**) at λ 254, 280, 350 nm.

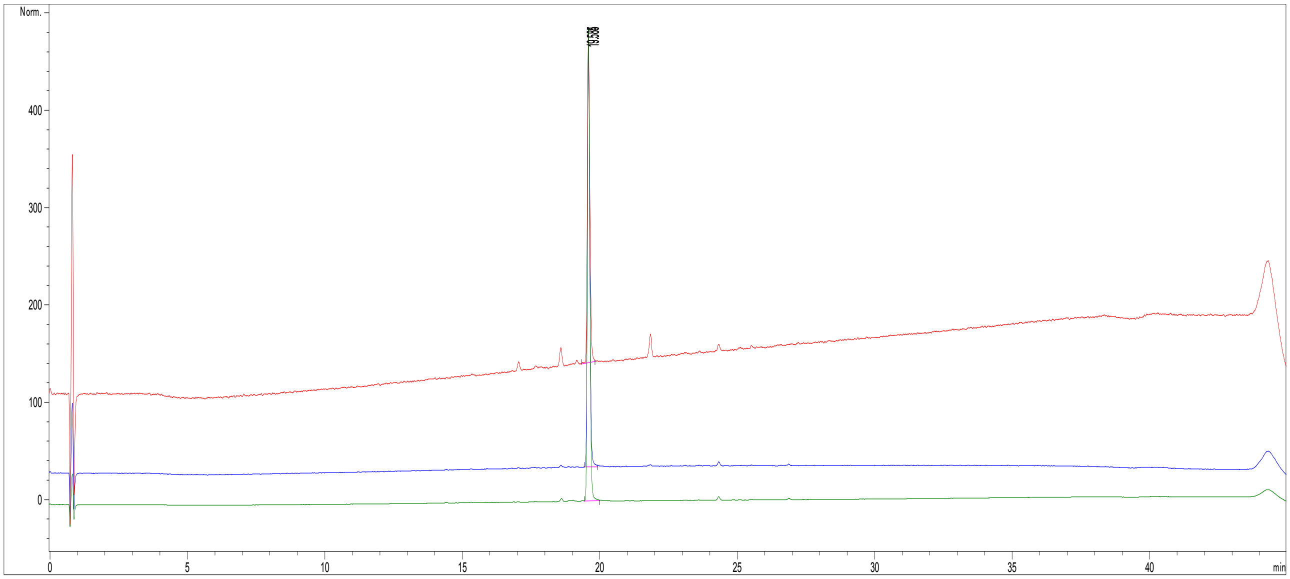

Figure S8. HPLC chromatogram of moracin C (**35**) at λ 254, 280, 350 nm.
